# Effect of polygenic scores on the relationship between psychosis and cognition

**DOI:** 10.1038/s41398-025-03666-z

**Published:** 2025-11-21

**Authors:** Lauren Varney, Krisztina Jedlovszky, Baihan Wang, Stephen Murtough, Marius Cotic, Alvin Richards-Belle, Noushin Saadullah Khani, Robin Lau, Rosemary Abidoph, Andrew McQuillin, Johan H. Thygesen, Psychosis Endophenotypes International Consortium (PEIC), Neeltje EM van Haren, Maria J. Arranz, Marta Di Forti, Ina Giegling, Cathryn Lewis, Kuang Lin, Andrew M. McIntosh, John Powell, Dan Rujescu, Matthias Weisbrod, Stephan Bender, Benedicto Crespo-Facorro, Jeremy Hall, Conrad Iyegbe, Eugenia Kravariti, Stephen M. Lawrie, Ignacio Mata, Colm McDonald, Robin M. Murray, Diana Prata, Timothea Toulopoulou, Elvira Bramon, Genetic Risk and Outcome of Psychosis (GROUP) Investigators, Therese van Amelsvoort, Wiepke Cahn, Lieuwe de Haan, Marieke van der Pluijm, Claudia J. P. Simons, Jim van Os, Wim Veling, Behrooz Z. Alizadeh, Behrooz Z. Alizadeh, Stephan Bender, Benedicto Crespo-Facorro, Jeremy Hall, Conrad Iyegbe, Eugenia Kravariti, Stephen M. Lawrie, Ignacio Mata, Colm McDonald, Robin M. Murray, Diana Prata, Timothea Toulopoulou, Neeltje EM van Haren, Elvira Bramon

**Affiliations:** 1 Division of Psychiatry, University College London, London, UK; 2 Division of Biosciences, University College London, London, UK; 3 Nuffield Department of Population Health, University of Oxford, Oxford, UK; 4 Department of Genetics and Genomic Medicine, UCL Great Ormond Street Institute of Child Health, University College London, London, UK; 5 Department of Psychology, Institute of Psychiatry, Psychology & Neuroscience, King’s College London, London, UK; 6 Institute of Health Informatics, Faculty of Population Health Sciences, University College London, London, UK; 7 Department of Epidemiology, University Medical Center Groningen, University of Groningen, Groningen, The Netherlands; 8 Department of Psychiatry, University Medical Center Groningen, University of Groningen, Groningen, The Netherlands; 9 University of Cologne, Faculty of Medicine and University Hospital Cologne, Department of Child and Adolescent Psychiatry, Psychosomatic Medicine and Psychotherapy, Cologne, Germany; 10 Centre for Biomedical Research in the Mental Health Network (CIBERSAM), Hospital Universitario Virgen del Rocío, Instituto de Biomedicina de Sevilla (IBIS)-CSIC, Seville, Spain; 11 Department of Psychiatry, University of Sevilla, Sevilla, Spain; 12 Division of Psychological Medicine and Clinical Neurosciences, Cardiff University, Cardiff, UK; 13 Neuroscience and Mental Health Innovation Institute, Cardiff University, Cardiff, UK; 14 Department of Genetics and Genomic Sciences, Icahn School of Medicine at Mount Sinai Hospital, New York, USA; 15 Department of Psychosis Studies, Institute of Psychiatry, Psychology and Neuroscience, King’s College London, London, UK; 16 Division of Psychiatry, University of Edinburgh, Edinburgh, UK; 17 Fundación Argibide, Pamplona (Navarra), Spain; 18 Clinical Science Institute, University of Galway, Galway, Ireland; 19 Centre for Neuroimaging & Cognitive Genomics (NICOG), Clinical Neuroimaging Laboratory, Galway Neuroscience Centre, College of Medicine Nursing and Health Sciences, University of Galway, H91 TK33 Galway, Ireland; 20 Institute of Psychiatry, Psychology and Neuroscience, King’s College London, London, UK; 21 Instituto de Biofísica e Engenharia Biomédica, Faculdade de Ciências da Universidade de Lisboa, Lisbon, Portugal; 22 Department of Psychology & National Magnetic Resonance Research Center (UMRAM), Aysel Sabuncu Brain Research Centre (ASBAM), Bilkent University, Ankara, Turkey; 23 Department of Psychiatry, Faculty of Medicine, National and Kapodistrian University of Athens, Athens, Greece; 24 Department of Psychiatry, Icahn School of Medicine at Mount Sinai, New York, NY USA; 25 Department of Child and Adolescent Psychiatry/Psychology, Erasmus Medical Centre, Rotterdam, Netherlands; 26 North London NHS Foundation Trust, London, UK; 27 Hospital Universitari MútuaTerrassa, Terrassa, Spain; 28 King’s College London, London, UK; 29 Medical University of Vienna, Vienna, Austria; 30 University of Oxford, Oxford, UK; 31 University of Edinburgh, Edinburgh, UK; 32 University of Heidelberg, Heidelberg, Germany; 33 Department of Psychiatry and Neuropsychology, Maastricht University Medical Center, School for Mental Health and Neuroscience, Maastricht, The Netherlands; 34 Brain Centre Rudolf Magnus, Department of Psychiatry, University Medical Center Utrecht, Utrecht University, Utrecht, The Netherlands; 35 Altrecht, General Mental Health Care, Utrecht, The Netherlands; 36 Department of Psychiatry, Amsterdam UMC, University of, Amsterdam, The Netherlands; 37 Arkin Institute for Mental Health, Amsterdam, The Netherlands; 38 GGzE Institute for Mental Health Care, Eindhoven, The Netherlands; 39 Department of Psychosis Studies, Institute of Psychiatry, King’s College London, King’s Health Partners, London, UK

**Keywords:** Predictive markers, Genetics

## Abstract

Cognitive impairment is an important but often under-researched symptom in psychosis. Both psychosis and cognition are highly heritable and there is evidence of a genetic effect on the relationship between them. Using samples of adults (*N* = 4 506) and children (*N* = 10 981), we investigated the effect of schizophrenia and bipolar disorder polygenic scores on cognitive performance, and intelligence and educational attainment polygenic scores on psychosis presentation. Schizophrenia polygenic score was negatively associated with visuospatial processing in adults (beta: −0.0569; 95% confidence interval [CI]: −0.0926, −0.0212) and working memory (beta: −0.0432; 95% CI: −0.0697, −0.0168), processing speed (beta: −0.0491; 95% CI: −0.0760, −0.0223), episodic memory (betas: −0.0581 to −0.0430; 95% CIs: −0.0847, −0.0162), executive functioning (beta: −0.0423; 95% CI: −0.0692, −0.0155), fluid intelligence (beta: −0.0583; 95% CI: −0.0847, −0.0320), and total intelligence (beta: −0.0458; 95% CI: −0.0709, −0.0206) in children. Bipolar disorder polygenic score was not associated with any cognitive domains studied. Lower polygenic scores for intelligence were associated with greater odds of psychosis in adults (odds ratio [OR]: 0.886; 95% CI: 0.811–0.968). In children, lower polygenic scores for both intelligence (OR: 0.829; 95% CI: 0.777–0.884) and educational attainment (OR: 0.771; 95% CI: 0.724–0.821) were associated with greater odds of psychotic-like experiences. Our findings suggest that polygenic scores for both cognitive phenotypes and psychosis phenotypes are implicated in the relationship between psychosis and cognitive performance. Further research is needed to determine the direction of this effect and the mechanisms by which it occurs.

## Introduction

Schizophrenia and bipolar disorder are two of the most prevalent disorders that fall under the broader category of “psychosis”, a term used to describe disorders characterised by alterations to an individual’s thoughts and/or perceptions [1]. They are also two of the most heritable psychiatric disorders, with heritability rates between 75–80% [2, 3] and a large overlap in the genetic variants associated with each [4].

Though the most common symptoms are hallucinations and delusions, impairments in cognitive functioning are also seen in the majority of individuals with psychosis [5]. This is an important yet often overlooked symptom as associations have been found between poorer cognitive functioning and poorer functional outcomes. Cognitive functioning is also heritable, with heritability estimates of approximately 50% [6], which appear to be similar in both people with psychosis and the general population [7, 8]. Cognitive impairments can also be seen in the unaffected relatives of those with psychosis [9–12] as well as individuals at clinical-high risk of psychosis [13, 14]. These factors have led to cognitive performance (both broadly and within individual cognitive domains) being proposed as an endophenotype for psychosis [15]. Endophenotypes are measurable traits that can be used to “bridge the gap” between the genetic components of a disorder and its symptom presentation and help understand the mechanisms that lead from genotype to phenotype [16, 17].

Meta-analytic evidence suggests that schizophrenia polygenic scores are significantly associated with overall cognitive performance in general population samples [18]. However, this pattern does not appear when samples are restricted to those with psychosis, possibly because the effect is already captured by diagnosis or simply due to the smaller sample sizes [19, 20]. Evidence is mixed on the exact components of cognitive ability affected by schizophrenia polygenic scores [21]. The strongest evidence is for a negative effect on performance IQ, attention, and premorbid intelligence [18], while verbal memory, crystalised intelligence, category fluency, and educational attainment show less association with the polygenic scores [22–25]. For bipolar disorder polygenic scores, the literature is even more mixed, with some evidence suggesting a negative effect on childhood executive functioning, performance IQ, and processing speed [26]. Other evidence has produced little association [19, 22, 27, 28], and some have found positive associations with creativity and educational attainment [29]. Therefore, there is room for further investigation into the exact components of cognitive ability that show associations with polygenic scores for schizophrenia and bipolar disorder.

Looking in the opposite direction, the polygenic scores for cognitive performance are associated with performance on cognitive tests in population samples [19, 30, 31], individuals with psychosis [19, 30–32], and ultra-high risk individuals [33], suggesting that cognition is influenced by similar genetic mechanisms irrespective of psychosis risk [19]. These polygenic scores have also been shown to be associated with psychosis presentation as polygenic scores for childhood intelligence [34], performance IQ [35], and general cognitive ability [36] found to be lower in individuals with psychosis compared to controls. This suggests that the effect of genetic factors on the relationship between psychosis and cognitive impairment may be bidirectional.

The majority of research on the relationship between psychosis and cognition has been conducted in adult samples, which is unsurprising given the age of onset for psychotic disorders. However, schizophrenia is a neurodevelopmental disorder [37, 38], cognitive impairments can be seen in the prodromal phase of psychosis [39–41], and the transition to psychosis is not typically associated with further cognitive decline [42, 43]. Therefore, extending this research beyond adulthood could add depth to our understanding of the genetic mechanisms behind the relationship between psychosis and cognitive performance. Psychotic-like experiences in childhood have been associated with poorer cognitive functioning [44, 45] and polygenic scores for cognitive performance and educational attainment have each been negatively associated with these psychotic-like experiences [46].

The aim of the present study was to examine this bidirectional effect of polygenic scores on the relationship between psychosis and cognitive performance in the same samples and in both adults and children. We examined the effect of polygenic scores for schizophrenia and bipolar disorder on performance within a range of individual cognitive domains as well as the effect of polygenic scores for cognitive performance and educational attainment on psychosis presentation.

## Method and materials

### Participants

#### Psychosis endophenotypes international consortium (PEIC)

PEIC is a collaborative effort from multiple sites across Europe (UK, the Netherlands, Spain, Germany) and Australia, comprising data from individuals with a diagnosis of psychosis (bipolar disorder, schizophrenia, or other psychotic disorder; hereafter patients), their unaffected relatives, and healthy control participants. All were of European ancestry [28, 47]. Relatives and controls were not excluded if they had a personal history of a non-psychotic psychiatric disorder, as long as they were off psychotropic medication for at least 12 months before assessment. Exclusion criteria (for all clinical groups) included a history of neurological disease or previous loss of consciousness due to head injury [9].

#### Adolescent brain cognition development (ABCD)® study

The ABCD Study® is a longitudinal study from the USA that aims to investigate the impact of various factors on brain development and health/social outcomes. A population-representative sample of children aged 9–10 years was recruited between 2016 and 2018 [48]. Participants were excluded if they were not fluent in English, had a history of traumatic brain injury, or a current diagnosis of moderate/severe autism spectrum disorder, schizophrenia, intellectual disability, or substance use disorders [49]. All data used here were taken from baseline assessments.

### Genotyping, quality control, and imputation

#### PEIC

DNA was extracted from blood samples of 6 935 participants and sent to the Wellcome Trust Sanger Institute (Cambridge, UK) for initial processing and quality control (QC). After imputation, 6 215 801 SNPs and 4 835 participants remained. Further details available in Bramon et al. [47] and Supplementary Information.

#### ABCD study^®^

DNA was extracted from blood/saliva samples of 11 880 participants at the Rutgers University Cell and DNA Repository (RUCDR; New Jersey, USA). After post-imputation QC, 11 229 083 SNPs and 11 017 participants remained. Further detail available in Uban et al. [50], Wang et al. [49] and Supplementary Information, as well at https://nda.nih.gov/study.html?id=901.

### Relationship inference and principal component analysis

#### PEIC and ABCD study^®^

The GENESIS R/Bioconductor package [51, 52] was used to account for familial relatedness and population structure. An unadjusted kinship matrix was generated first using KING-robust 2.2.5 [53] to infer the relatedness of each pair of participants. The SNPRelate package in R 4.0.2 [54] was used to analyse the genotyped data alongside this kinship matrix to estimate ancestrally representative principal components (PCs). An adjusted kinship matrix was then generated to account for these PCs, allowing for an estimation of familial relatedness independent of ancestry. Further details available in Supplementary Information and Wang et al. [49].

### Polygenic score generation

All polygenic scores were generated using PRS-CSx [55]. Reference panels from the 1000 Genomes Project [56] that best matched the ancestries present in the original genome-wide association studies (GWAS) were used (bipolar disorder, educational attainment, and intelligence polygenic scores: European panel; schizophrenia polygenic score: European, East Asian, African, and Admixed American panels; see Supplementary Information). Scores were standardised against the sample mean (in the PEIC sample, the control group mean).

For schizophrenia and bipolar disorder polygenic scores, summary statistics from the Psychiatric Genomic Consortium (PGC) analyses were used [57, 58]. As the PEIC data was used in the PGC schizophrenia discovery sample, summary statistics were obtained that excluded this sample to avoid overlap. For educational attainment polygenic scores, summary statistics from Lee et al. [59] were used. For the intelligence polygenic scores, summary statistics from Savage et al. [60] were used.

### Cognitive tests

#### PEIC

Three tests were administered: block design and digit span from the Wechsler Adult Intelligence Scale, revised version (WAIS-R) [61] or third edition (WAIS-III) [62], and the Rey Auditory Verbal Learning Test (RAVLT) [63].

#### ABCD study^®^

Participants completed 11 neurocognitive tests, seven from the National Institutes of Health (NIH) Toolbox^®^ (Picture Vocabulary; Oral Reading Recognition; Pattern Comparison; List Sorting; Picture Sequence; Flanker; Dimensional Change Card Sort) and four additional tests (RAVLT; Cash Choice Task; Little Man Task; Matrix Reasoning) [64].

The NIH Toolbox^®^ tests create three composite scores: Crystalised Intelligence, Fluid Intelligence, and Total Intelligence [64]. The cognitive domains that each test measures and further detail on each test is available in Supplementary Information.

### Psychosis outcome

#### PEIC

All participants underwent a structured clinical interview with a psychiatrist to confirm (for patients) or rule out (for relatives and controls) the presence of a DSM-IV diagnosis of schizophrenia or another psychotic disorder [28, 47]. This led to three clinical groups: patients, relatives, and controls. These groups were used as the psychosis outcome in the adult sample.

#### ABCD study^®^

Responses on the Prodromal Questionnaire–Brief Child Version (PQ-BC), a 21-item self-report questionnaire of psychotic-like experiences in the past month, were used to measure psychotic-like experiences. Each item has three parts: whether they experienced the symptom; if yes, whether it was distressing; and, if yes, how distressing on a scale of 1 (“not very bothered”) to 5 (“extremely bothered”). Scores of 3 (“moderately bothered”) or more were classed as significantly distressing [45]. Three levels of psychotic-like experiences were created as the outcome in the child sample, increasing in severity: psychotic-like experiences; distressing psychotic-like experiences; and significantly distressing psychotic-like experiences.

### Statistical analyses

After removing participants with incomplete data, 4 506 participants remained in the PEIC sample and 10 981 remained in the ABCD Study^®^ sample. Mixed model regression analyses (linear/logistic) were used to investigate the effect of polygenic scores on cognitive performance and psychosis presentation. Age, sex, and ancestry PCs were included as fixed effects (as well as research site for all PEIC analyses, and clinical group in the cognitive performance PEIC analyses); the adjusted kinship matrix was included as a random effect. Linear cognitive test scores were standardised against the group mean in the ABCD Study^®^ sample analyses and against the control group mean in the PEIC sample analyses. Interaction and subgroup analyses were also conducted within the PEIC sample to determine whether the effect of polygenic scores differed between the clinical groups.

A multiple testing correction of 0.05/4 (three cognitive tests and group status prediction) was applied to the analyses carried out with the PEIC sample, leaving an adjusted significance threshold of *p* < 0.0125. In the ABCD Study^®^ dataset, cognitive tests were grouped by domain. This resulted in seven domains (Supplementary Table S1) which, combined with the psychotic-like experience prediction, lead to an adjusted significance threshold of *p* < 0.00625 (0.05/8). Uncorrected *p*-values are reported but interpreted using the respective adjusted threshold.

We also used a Support Vector Machine (SVM) supervised machine learning algorithm to predict the group status in both the adult sample (patient, relative, or control) and child sample (psychotic-like experience groups) from cognition-related polygenic scores and demographic parameters (age, sex, ancestry PCs, research site), following the method from Bracher Smith et al. [65]. This was included as a robust and replicable baseline to validate and benchmark the regression. To assess the relative importance of the polygenic scores compared to demographic predictors on clinical group status, the SVM model was further inspected using permutation feature importance [66]. Further details of these analyses can be found in the Supplementary Information.

#### PEIC

Participants gave written informed consent and Institutional Review Board (IRBs) at each collecting site approved the study. Laboratory and data analyses across sites were approved under the following IRBs: The UK National Health Service Multicentre Research Ethics Committee (19/LO/1403 and 03/11/090); and the Ethics Committee at the Institute of Psychiatry, Psychology and Neuroscience at King’s College London (references 038/00 and 011/99).

#### ABCD study^®^

Informed assent/consent was obtained from participants and their parents at the research centre they were recruited at. Ethical approval of the research protocol was provided for most sites by a central IRB at the University of California, San Diego (IRB# 160091); some sites obtained local IRB approval [67].

## Results

### Demographics

Demographic data for participants in the PEIC sample are presented in Table 1 and for the ABCD Study^®^ sample in Table 2. In both samples, the split between males and females was relatively equal (though in the PEIC sample subgroups, there were more male patients and more female relatives). In the ABCD sample, just over half were of White ethnicity.

**Table 1:** Demographic Characteristics for the Adult (PEIC) Sample.

	Patients	Relatives	Controls	Total
**Sample size**, N	1182	854	2470	4506
**Age**, mean years (SD)	33.5 (10.4)	45.7 (15.9)	45.5 (16.2)	42.4 (15.8)
Age range	15–79	16–85	16–84	15–85
**Gender**, n (%)				
Male	794 (67.2%)	344 (40.3%)	1182 (47.9%)	2320 (51.5%)
Female	388 (32.8%)	510 (59.7%)	1288 (52.1%)	2186 (48.5%)
**Centre**, n (%)				
Edinburgh	31 (2.6%)	–	17 (<0.1%)	48 (1.1%)
Heidelberg	24 (2.0%)	8 (0.9%)	22 (0.9%)	55 (1.2%)
Holland	370 (31.3%)	505 (59.1%)	974 (39.4%)	1849 (41.0%)
London	237 (20.0%)	197 (23.1%)	324 (13.1%)	758 (16.8%)
Munich	–	–	962 (38.9%)	962 (21.3%)
Pamplona	44 (3.7%)	–	–	44 (1.0%)
Perth	309 (26.1%)	143 (16.7%)	163 (6.6%)	615 (13.6%)
Santander	167 (14.1%)	–	8 (0.3%)	175 (3.9%)
**Diagnosis**, n (%)				
Anxiety and/or Depressive Disorder	–	192 (22.5%)	173 (7.0%)	365 (8.1%)
Bipolar Disorder	107 (9.1%)	–	–	107 (2.4%)
Other Psychotic Disorder	169 (14.3%)	–	–	169 (3.8%)
Personality Disorder	–	1 (0.1%)	–	1 (<0.1%)
Schizophrenia	906 (76.6%)	–	–	906 (20.1%)
Substance Misuse	–	4 (0.5%)	11 (0.4%)	15 (0.3%)
No Psychiatric Disorder	–	657 (76.9%)	2286 (92.6%)	2943 (65.3%)

*PEIC* psychosis endophenotypes international consortium, *N/n* number of participants, *SD* standard deviation.

**Table 2:** Demographic Characteristics for the Child (ABCD Study^®^) Sample.

	All participants
**Sample size**, N (%)	10,981
**Age**, years (mean, SD)	9.9 (0.63)
Age range, years	8.9–11.1
**Gender**, n (%)	
Male	5805 (52.9%)
Female	5176 (47.1%)
**Ethnicity**, n (%)	
White	5872 (53.5%)
Black	1684 (15.3%)
Hispanic	2099 (19.1%)
Asian	155 (1.4%)
Other	1169 (10.6%)
*Missing*	*2 (*<*0.1%)*
**Psychotic-Like Symptoms (PQ-B)**, n (%)	
At Least One Symptom	6735 (61.3%)
Distress From At Least One Symptom	4748 (43.2%)
Significant Distress From At Least One Symptom	2931 (26.7%)
*Missing*	*5 (*<*0.1%)*

*ABCD* adolescent brain cognitive development, *N/n* number of participants, *SD* standard deviation, *PQ-B* prodromal questionnaire – brief version.

### Cognitive test performance

Details of average cognitive performance for both the PEIC sample and ABCD Study^®^ sample are available in the Supplementary Information and Supplementary Tables S2–S4. Correlation matrices displaying the correlation between scores on the different tests included in the study are displayed in Supplementary Figure S1 (PEIC sample) and Supplementary Figure S2 (ABCD Study^®^ sample).

### Effect of polygenic scores on cognitive performance

#### Effect of polygenic scores on cognitive performance: PEIC (Adult) sample

**Fig. 1:**
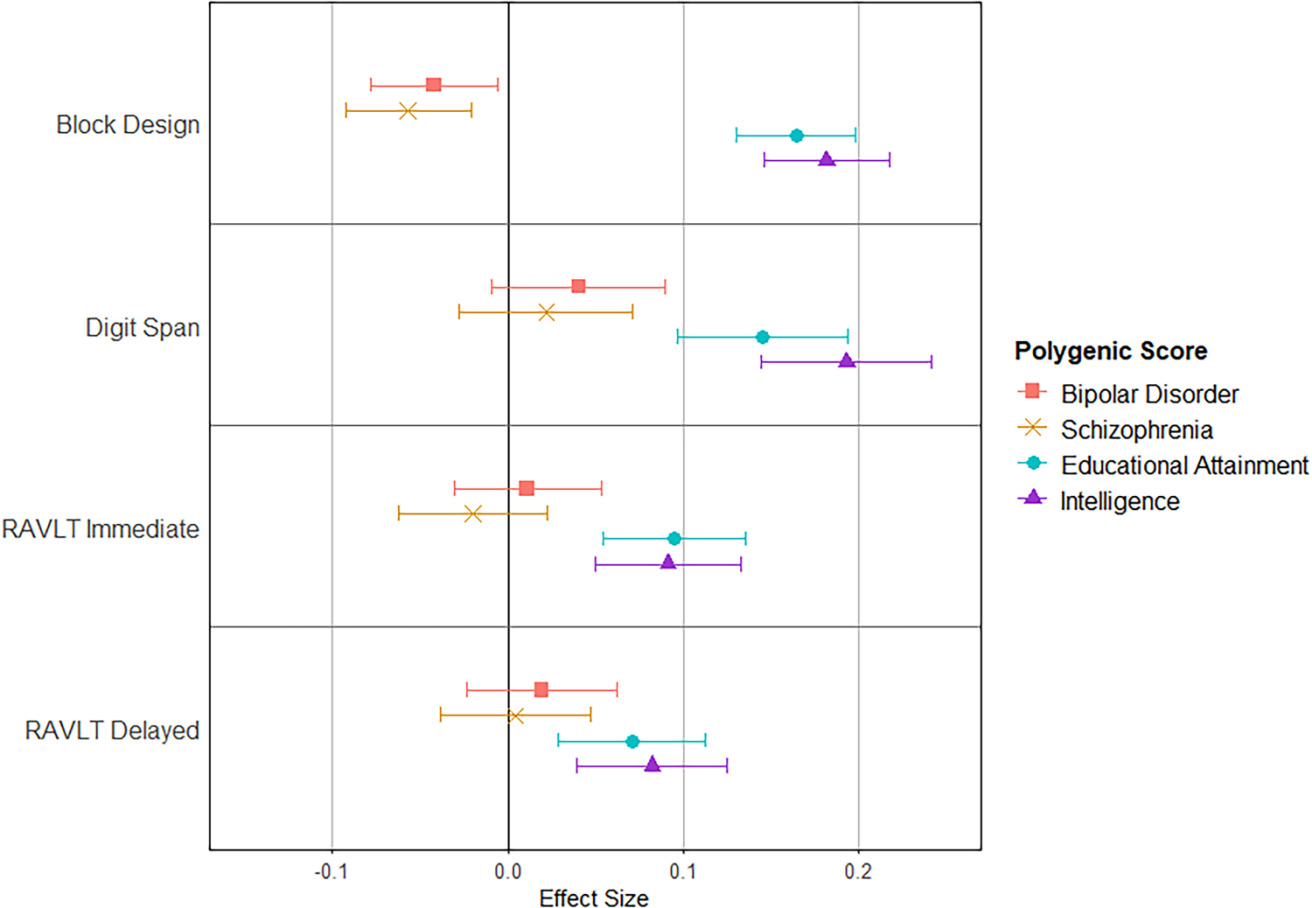
Effect of Polygenic Scores on Cognitive Test Performance in the PEIC Sample. Effect of psychosis-related and cognition-related polygenic scores on cognitive test performance in adults, while controlling for the effect of age, gender, clinical group (patient/relative/control), research site, ancestry (the first four ancestry principal components), and participant inter-relatedness (kinship matrix). Scores have been standardised using the mean and standard deviation from the control group. Standardised values are given in Supplementary Table S5; non-standardised values are given in Supplementary Table S6. PEIC Psychosis Endophenotype International Consortium, RAVLT Rey Auditory Verbal Learning Task.

Schizophrenia polygenic score was negatively associated with block design performance (mean difference: -0.0569; 95% confidence interval [CI]: −0.0926, −0.0212; *p* = 0.00179), but no associations were identified with other cognitive tests (*p* values > 0.356). Bipolar disorder polygenic scores showed no significant associations after multiple testing correction (*p* values > 0.0219). Intelligence (*p* values < 0.000165) and educational attainment (*p* values < 0.000897) polygenic scores were each significantly positively associated with performance on all tests (Fig. 1; Supplementary Tables S5 and S6).

Interaction analyses between polygenic scores and clinical group were non-significant, suggesting no difference in the effect of the polygenic scores between the clinical groups. Interaction and subgroup analyses are discussed in detail in Supplementary Tables S7, S8, S17, and S18 and the Supplementary Information.

#### Effect of polygenic scores on cognitive performance: ABCD study^®^ (Child) sample

**Fig. 2:**
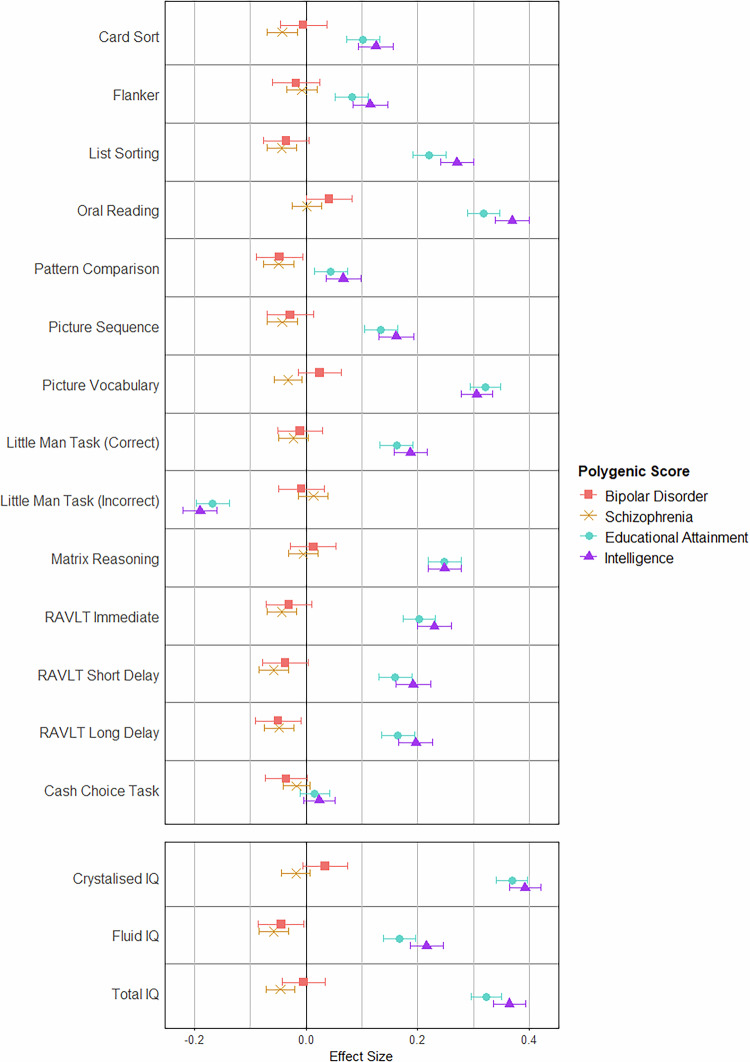
Effect of Polygenic Scores on Performance on Individual Cognitive Tests and Composite Intelligence Scores in the ABCD Study^®^ Sample. Effect of psychosis-related and cognition-related polygenic scores on cognitive test performance in children, while controlling for the effect of age, gender, ancestry (the first eight ancestry principal components), and participant inter-relatedness (kinship matrix). Scores have been standardised using the mean and standard deviation from the whole sample. Standardised values are given in Supplementary Table S9 and S11; non-standardised values are given in Supplementary Table S10 and S12. *Note:* “Little Man Task (Correct)” refers to the percentage of participants’ responses were correct during the task. “Little Man Task (Incorrect)” refers to the percentage of participants’ responses were incorrect. Cash Choice Task effect is the log-transformed result from a logistic regression analysis. Effect > 0 indicates greater odds of choosing the delayed gratification option ($115 in three months); effect < 0 indicates greater odds of choosing the immediate gratification option ($75 in three days). Log-transformed values are given in Supplementary Table S13; logistic regression results are given in Supplementary Table S14. ABCD Adolescent Brain Cognitive Development, RAVLT Rey Auditory Verbal Learning Task.

Schizophrenia polygenic score was negatively associated with Fluid Intelligence (mean difference: −0.0583; 95% CI: −0.0847, −0.0320; *p* = 1.44 × 10^−5^) and Total Intelligence (mean difference: −0.0458; 95% CI: −0.0709, −0.0206; *p* = 0.000362) composite scores, but not Crystalised Intelligence composite scores (*p* = 0.162). For individual tests, higher schizophrenia polygenic score was associated with lower scores on: Dimensional Change Card Sort (mean difference: −0.0423; 95% CI: −0.0692, −0.0155, *p* = 0.00201), List Sorting (mean difference: −0.0432; 95% CI: −0.0697, −0.0168; *p* = 0.00136), Pattern Comparison (mean difference: −0.0491; 95% CI: −0.0760, −0.0223; *p* = 0.000342), and Picture Sequence (mean difference: −0.0430; 95% CI: −0.0697, −0.0162; *p* = 0.00164) from the NIH Toolbox^®^ (four of the five tests that comprise the Fluid Intelligence composite score), as well as immediate (mean difference: −0.0437, 95% CI: −0.0699, −0.0174; *p* = 0.00111), short-delayed (mean difference: −0.0581; 95% CI: −0.0847, −0.0315; *p* = 1.92 × 10^−5^), and long-delayed (mean difference: −0.0483; 95% CI: −0.0750, −0.0216; *p* = 0.000397) recall on the RAVLT (Fig. 2).

There was no significant effect of bipolar disorder polygenic score on performance in any of the cognitive tests or composite scores after correction for multiple comparisons (*p* values > 0.0185). Intelligence (*p* values < 2.71 × 10^−5^) and educational attainment (*p* values < 0.00381) polygenic scores were significantly associated with performance on all tests and composites. Full results are presented in Supplementary Tables S9–S14.

### Effect of polygenic scores on psychosis presentation

#### Effect of polygenic scores on psychosis presentation: PEIC (Adult) sample

**Fig. 3:**
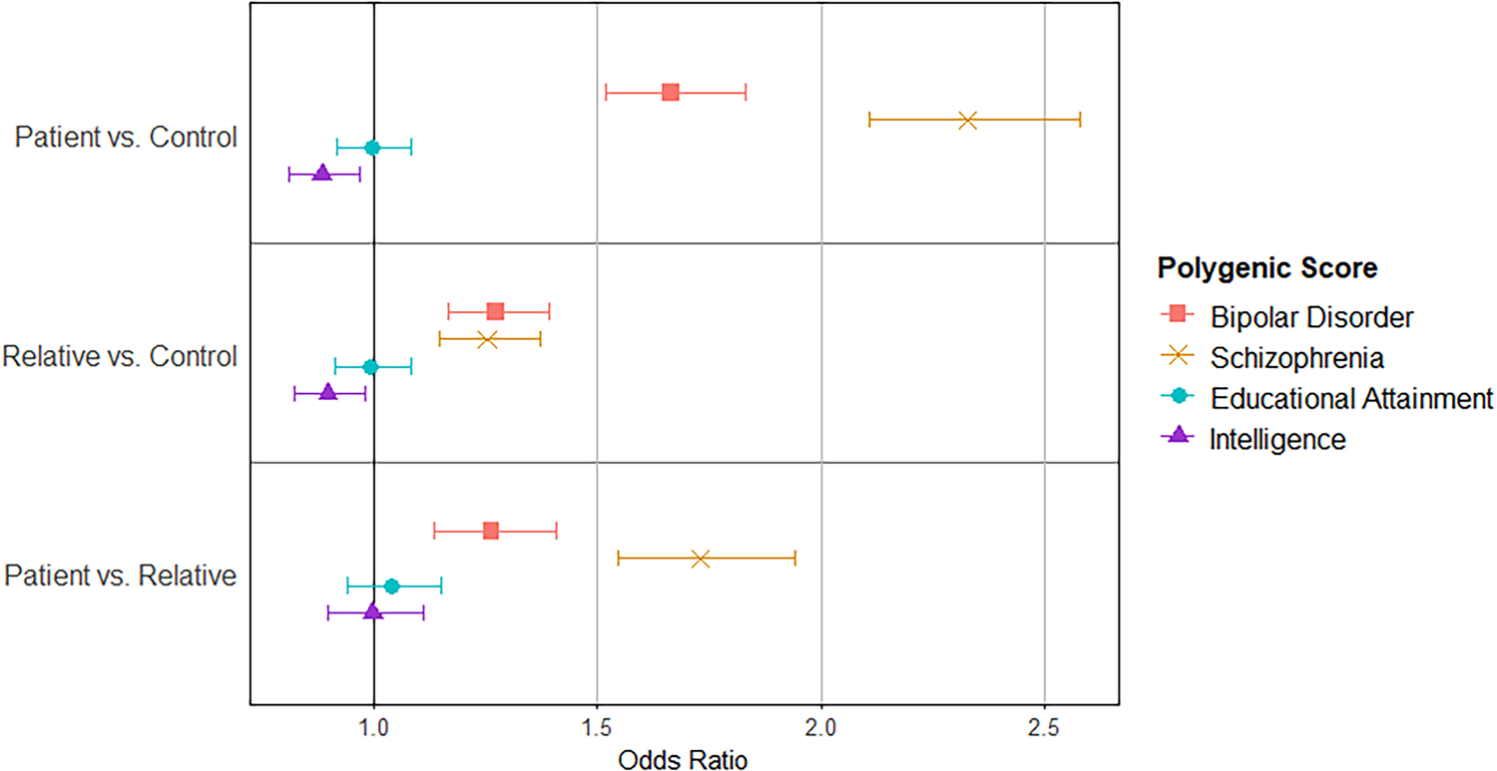
Prediction of Clinical Group Status Using Cognition-Related Polygenic Scores in the PEIC Sample. Effect of psychosis-related and cognition-related polygenic scores on the odds of being in the comparison group (i.e. patient, relative, patient, respectively from top to bottom) compared to the odds of being in the comparison group (i.e. control, control, relative, respectively). Values are given in Supplementary Table S15. PEIC Psychosis Endophenotypes International Consortium.

The logistic regression models showed that intelligence polygenic score was able to distinguish between patients and controls (odds ratio [OR]: 0.886; 95% CI: 0.811–0.968; *p* = 0.00719). There was also a trend towards distinguishing between relatives and controls (*p* = 0.0190; Fig. 3), but this did not pass the adjusted significance threshold (*p* < 0.0125). Educational attainment polygenic scores was a poor predictor of clinical group in the adult sample (*p* values > 0.445; Supplementary Table S15).

Both bipolar disorder polygenic scores (*p* values < 2.38 × 10^−5^) and schizophrenia polygenic scores (*p* values < 6.80 × 10^−7^) were able to significantly distinguish between all clinical groups.

In the machine learning analysis, while the SVM models including intelligence polygenic scores and educational attainment polygenic scores were each able to distinguish between patients and controls (median area under the receiver operating characteristic [AUROC_median_]: 0.847 and AUROC_median_: 0.856, respectively) and between patients and relatives (AUROC_median_: 0.781 and AUROC_median_: 0.752, respectively) with high accuracy, the relative importance of the polygenic scores was low. This suggests the model’s performance was driven by the covariates included in the model. Further details available in the Supplementary Information.

#### Effect of polygenic scores on psychosis presentation: ABCD Study^®^ (Child) sample

**Fig. 4:**
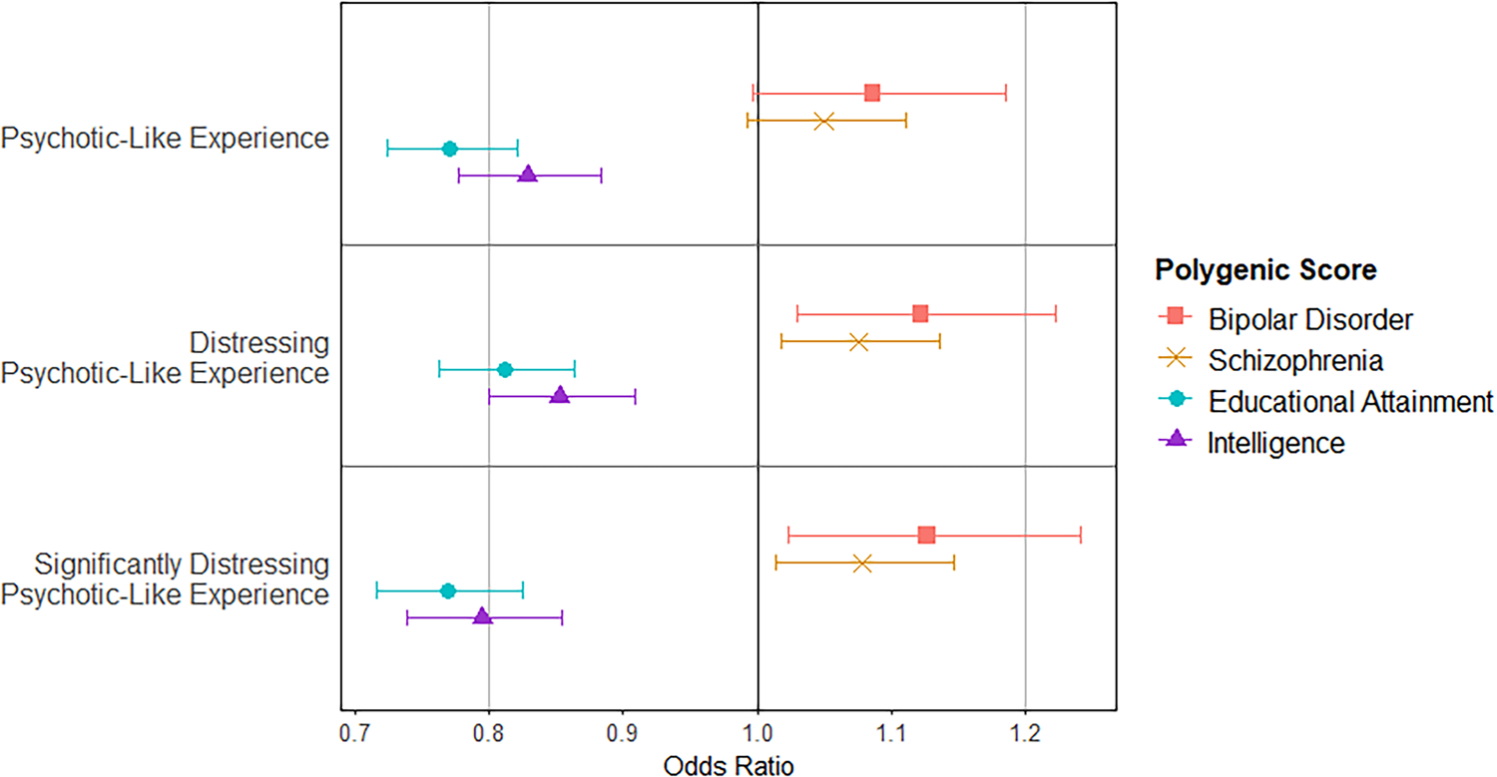
Prediction of Psychotic-Like Experiences at Baseline Using Polygenic Scores in the ABCD Study^®^ Sample. Effect of psychosis-related and cognition-related polygenic scores on the odds of experiencing at least one of the given types of psychotic-like experiences (i.e. odds of experiencing at least one psychotic-like experience, at least one distressing psychotic-like experience, at least one significantly distressing psychotic-like experience, respectively from top to bottom) compared to the odds of not having such experiences. Psychotic-like experiences measured using the Prodromal Questionnaire–Brief Child Version (PQ-BC). Values given in Supplementary Table S16. ABCD Adolescent Brain Cognitive Development.

The regression models showed that educational attainment polygenic score significantly distinguished between children who experienced psychotic-like experiences at baseline and those who did not (OR: 0.771; 95% CI: 0.724–0.821; *p* = 5.86 × 10^−16^), those who experienced distressing psychotic-like experiences and those who did not (OR: 0.813, 95% CI: 0.764–0.864, *p* = 5.48 × 10^−11^), and those who experienced significantly distressing psychotic-like experiences and those who did not (OR: 0.769; 95% CI: 0.717–0.826; *p* = 3.87 × 10^−13^) (Fig. 4 and Supplementary Table S16). Educational attainment polygenic score was also significantly negatively associated with the number of each category of psychotic-like experiences reported (*p* values < 2.52 × 10^−11^; Supplementary Table S16).

Intelligence polygenic score was also negatively associated with all psychotic-like experience outcomes in the regression models. A greater intelligence polygenic score was associated with decreased odds of psychotic-like experiences (OR: 0.829; 95% CI: 0.777–0.884; *p* = 1.35 × 10^−8^), distressing psychotic-like experiences (OR: 0.853; 95% CI: 0.800–0.909; *p* = 1.02 × 10^−6^), and significantly distressing psychotic-like experiences (OR: 0.795; 95% CI: 0.740–0.855; *p* = 6.32 × 10^−10^) (Fig. 4), as well as the number of each type of psychotic-like experience reported (*p* values < 3.83 × 10^−6^; Supplementary Table S16).

For both schizophrenia and bipolar disorder polygenic scores, there was only one significant result after correcting for multiple comparisons. In both cases, this was the distinction between those who experienced distressing psychotic-like experiences and those who did not. Higher schizophrenia polygenic scores (OR: 1.08; 95% CI: 1.02–1.14; *p* = 0.00939) and bipolar disorder polygenic scores (OR: 1.12; 95% CI: 1.03, 1.22; *p* = 0.00817) were associated with increased odds of reporting such experiences.

When the group classification analyses were carried out using the full SVM model, each performed at near the 0.5 (chance) level (AUROC: 0.550–0.613). Further detail available in the Supplementary Information.

## Discussion

We aimed to examine the effect of polygenic scores for both schizophrenia and bipolar disorder on cognitive performance within specific cognitive domains, examine the effect of polygenic scores for intelligence and educational attainment on psychosis presentation, and extend previous research in this area by exploring these associations in both adults and children. Our results suggest that schizophrenia polygenic scores and bipolar disorder polygenic scores have different effects on cognitive performance, and that these effects differ across cognitive domains and possibly across different stages of life. In terms of cognition-related polygenic scores, only intelligence polygenic scores were able to distinguish between clinical groups (psychosis cases, unaffected relatives, and healthy controls) in the adult sample, while both the educational attainment and intelligence polygenic scores distinguished between the psychotic-like experience groups in the child sample. However, when these group distinction analyses were carried out using the machine learning model, the relative importance of the polygenic scores was low, suggesting their impact on prediction is minimal.

### Influence of polygenic scores for psychosis on cognitive performance

Our evidence supports previously reported negative associations between schizophrenia polygenic scores and cognitive performance [18, 33, 34]. This effect appeared to differ both between adults and children and between the different cognitive domains. In the adult sample, higher schizophrenia polygenic scores were associated with poorer performance on the visuospatial processing task (block design), but not with the tests of working memory or episodic memory. In the child sample, higher schizophrenia polygenic scores were associated with poorer language ability, working memory, processing speed, and episodic memory, while there was no evidence of association with visuospatial processing. Methodological factors may have contributed to this difference (e.g. differences in the tests used, different sample sizes, controlling for clinical group in the PEIC analyses), but there is a possibility these findings represent a difference in the effect of schizophrenia risk over time or distinct maturational paths of the individual cognitive domains. There is conflicting evidence on the effect of schizophrenia polygenic scores on the domains that differed between the adult and child samples [23, 35, 68, 69], suggesting further research is needed to pinpoint these associations.

In both samples, we found limited evidence for an association between bipolar disorder polygenic scores and cognitive performance, in line with previous evidence [22, 26–28, 70]. One test showed a stronger association with bipolar disorder polygenic scores than schizophrenia polygenic scores: the Cash Choice task (though this result did not pass the adjusted significance threshold). Bipolar disorder polygenic score was associated with greater odds of choosing the immediate gratification option ($75 in three days) compared to delayed gratification ($115 in three months). This suggests greater impulsivity, which has been linked with bipolar disorder specifically [71, 72] so may serve as a mania/bipolar disorder-specific marker, though some evidence suggests it is associated with psychosis more generally [73].

While these findings replicate associations found in other samples, they cannot provide detail on the causal relationship between schizophrenia polygenic scores and cognitive performance. While a direct effect is possible, it may also be that the schizophrenia-associated genetic variants sensitise the individual to other factors (genetic or non-genetic) that then lead to cognitive impairment [43]. Further research that specifically aims to determine the causality of this relationship is needed to increase our understanding.

### Influence of polygenic scores for cognition on psychosis presentation

Intelligence polygenic scores showed a stronger association with psychosis outcomes in both adults and children compared to educational attainment polygenic scores. Intelligence polygenic score distinguished adult patients from controls, though with a smaller effect size than schizophrenia or bipolar disorder polygenic scores. There was also a trend towards distinguishing relatives from controls, though this did not pass the adjusted significance threshold for multiple comparisons. The odds ratio for distinguishing between patients and relatives, however, was almost exactly 1, suggesting that in terms of intelligence polygenic score, relatives and patients are more alike compared to controls. This differs from the schizophrenia polygenic score, which showed a greater effect size in distinguishing between patients and relatives compared to relatives and controls. Intelligence polygenic score also distinguished each category of psychotic-like experience in the child sample, while both the schizophrenia and bipolar disorder polygenic scores were each only able to distinguish one category (distressing psychotic-like experiences) and with smaller effect sizes. Educational attainment polygenic score was also negatively associated with all categories of psychotic-like experiences in the child sample, but did not distinguish between any of the clinical groups in the adult sample. This aligns previous findings that polygenic scores for cognitive performance/intelligence are more associated with schizophrenia case-status than educational attainment polygenic scores [30, 31, 45], possibly due to the non-genetic factors also involved in educational attainment [46, 74, 75]. However, the variance explained in psychosis outcomes was extremely small. Also, while the effect of the polygenic scores in the regression models, controlling for covariates, was significant, the machine learning analyses did not find the polygenic scores to contribute considerably in the adult sample and analyses performed at chance level in the child sample. While there may be a statistically significant effect of cognition-related polygenic scores on psychosis presentation, the clinical implications may be minimal.

We found a changing pattern of associations with psychosis presentation, whereby cognition-related polygenic scores were more strongly associated with psychotic-like experiences in childhood than psychosis-related polygenic scores, but psychosis-related polygenic scores were more strongly associated with case-control status in adulthood than the cognition-related polygenic scores. This raises questions about the similarities and differences between cognition-related and psychosis-related polygenic scores, the pathways through which they exert their phenotypic effects, and how this may differ across the lifespan. However, caution should also be taken when interpreting the associations in the child sample, as the differences in results may also be due to different phenotype classification. Although the Prodromal Questionnaire–Brief Child Version (PQ-BC) has been validated as a measure of early risk for psychosis [76], these symptoms are not necessarily indicative of psychosis in adulthood [77]. In the child sample, the proportion that reported experiencing at least one psychotic-like experience was well over the proportion expected to develop psychosis (and it should be noted that a formal, current diagnosis of schizophrenia was an exclusion criterion in the ABCD Study^®^, though the prevalence within this age group would be expected to be very low). This may be a factor in the difference in effect of the cognition-related polygenic scores in the child and adult samples. Childhood psychotic-like experiences may instead be indicative of an increased risk of later psychopathology more generally [78] rather than a predictor of those who will go on to meet criteria for a psychotic disorder. Cognition-related (particularly intelligence) polygenic scores may be able to provide an early indicator of who may continue experiencing such symptoms and develop psychosis in adulthood.

The mechanisms by which cognition-related polygenic scores are associated with psychosis may be rooted in the individual’s cognitive reserve [79]. Those with lower cognition-related polygenic scores may have a reduced cognitive reserve, which may affect a range of factors should they develop symptoms of psychosis in adulthood. Cognition-related polygenic scores (particularly those for cognitive performance/intelligence) may therefore act as a moderator on the relationship between genetic risk for psychosis and symptom presentation in adulthood. In a similar way, the polygenic score for schizophrenia resilience (common variants that may ameliorate the effect of schizophrenia risk variants in the context of a high schizophrenia polygenic risk score [80]) has been positively associated with cognitive performance across a range of cognitive domains [81], suggesting the variants that appear to have a protective effect on schizophrenia development may exert their effect, at least in part, through cognitive pathways.

### Limitations

While we made efforts to increase the ancestry diversity by using all participants in the ABCD Study^®^, the accuracy of the polygenic score calculations are limited by the GWAS discovery samples, which remain mostly European [57–60]. Efforts are in place to carry out GWASs in global populations beyond Europe [82–84]. Methods of generating polygenic scores that allow summary statistics from multiple ancestries to be used together, such as PRS-CSx, also help to improve accuracy when GWAS summary statistics from multiple ancestry groups are available [55, 85, 86]. However, without diversity in the first step of the process (i.e. the discovery GWAS samples), polygenic scores will remain less accurate in non-European ancestry groups.

Another limitation is that polygenic scores only account for common variants associated with an outcome. The SNP heritability for schizophrenia, bipolar disorder, and intelligence, are each reported as around 20% [57, 58, 60], despite overall heritability rates of 60–80% for schizophrenia [57], 60–85% for bipolar disorder [58], and 50% for intelligence [60], which suggests that not all of the heritability is being accounted for by the common variants identified in GWAS. Other genetic factors, including copy number variants (CNVs), are known to be associated with both psychosis and cognitive functioning [87, 88]. The use of microarray methods to genotype GWAS participants has also been suggested to play a role in the “missing heritability”; it is hoped that moving towards whole genome sequencing approaches may reduce this [89].

In our adult sample, individuals with a diagnosis of bipolar disorder (9% of the patient group) must have also experienced psychosis to be included in the cohort. These individuals may represent a more homogenous and severe subset that may be more similar, phenotypically and potentially genetically, to those with schizophrenia compared to the overall bipolar disorder population. This, as well as the low proportion of those with bipolar disorder compared to schizophrenia, may partially explain the stronger associations between schizophrenia polygenic scores and outcomes in this sample compared to bipolar disorder polygenic scores.

### Implications

These results, if replicated, add to the growing evidence for a genetic component in the relationship between psychosis and cognitive impairment [18]. As cognitive impairment in psychosis is associated with poorer health outcomes [5, 90, 91], such evidence may help to identify those at risk. Future research should prioritise using samples of different ancestry groups. As polygenic profiling becomes more widely available, without these efforts, such techniques may further exacerbate health disparities/inequity instead of improving healthcare [92, 93].

Finally, longitudinal studies such as the ABCD Study^®^ provide the opportunity for developmental research in this field. As participants will be followed up for up to 10 years (from age 9–10 until 19–20), patterns in both cognitive performance and psychosis presentation can be followed across the developmental period. This timeframe also covers the period previously used to examine the change in heritability of cognitive functioning [94], meaning this sample could be used to examine whether this change affects the impact of genetic factors on the relationship between psychosis and cognition.

### Conclusion

We found evidence that both cognition-related and psychosis-related polygenic scores are implicated in the association between psychosis and cognitive functioning, and that this can be seen in both adults and children. Cognition-related polygenic scores were more strongly associated with psychotic-like experiences in childhood than psychosis-related polygenic scores, but this association reversed when distinguishing adults with psychosis from controls, suggesting the contribution of genetic variants associated with each phenotype on the presentation of psychosis may differ over the life course. Further research is needed to pinpoint the causal effects in these relationships and the mechanisms through which they occur.

## Supplementary information

Supplementary Material

Supplementary Tables S2-S20

## Data Availability

The data used to generate the results are available upon request.

## Code availability

The code used to generate the results is available upon request.
